# Rectal Troubles

**Published:** 1889-10

**Authors:** 


RECTAL TROUBLES.—See the card of Dr. K. C. Divine in this journal. The Doctor has acquired a reputation in the treatment of rectal troubles. This is a class of diseases that can but be best treated by specialists prepared with suitable instruments and appliances for the purpose. It is well for the busy practitioner, and often very desirable, to refer troublesome cases in this line to a reliable specialist. We feel safe in recommending Dr. Divine to the profession in this department of practice.

